# Smartphone-Enabled Personalized Diagnostics: Current Status and Future Prospects

**DOI:** 10.3390/diagnostics11061067

**Published:** 2021-06-09

**Authors:** Karla Jaimes Merazzo, Joseba Totoricaguena-Gorriño, Eduardo Fernández-Martín, F. Javier del Campo, Eva Baldrich

**Affiliations:** 1 Basque Center for Materials, Applications and Nanostructures, UPV/EHU Science Park, 48940 Leioa, Spain; karla.merazzo@bcmaterials.net (K.J.M.); joseba.totoricaguena@bcmaterials.net (J.T.-G.); eduardo.fernandez@bcmaterials.net (E.F.-M.); 2 IKERBASQUE, Basque Foundation for Science, 48009 Bilbao, Spain; 3 Diagnostic Nanotools Group, CIBBIM-Nanomedicine, Vall d’Hebron Institut de Recerca (VHIR), Vall d’Hebron Barcelona Hospital Campus, 08035 Barcelona, Spain

**Keywords:** point of care devices, smarthphone-based diagnostics, molecular methods, biosensors

## Abstract

Smartphones are becoming increasingly versatile thanks to the wide variety of sensor and actuator systems packed in them. Mobile devices today go well beyond their original purpose as communication devices, and this enables important new applications, ranging from augmented reality to the Internet of Things. Personalized diagnostics is one of the areas where mobile devices can have the greatest impact. Hitherto, the camera and communication abilities of these devices have been barely exploited for point of care (POC) purposes. This short review covers the recent evolution of mobile devices in the area of POC diagnostics and puts forward some ideas that may facilitate the development of more advanced applications and devices in the area of personalized diagnostics. With this purpose, the potential exploitation of wireless power and actuation of sensors and biosensors using near field communication (NFC), the use of the screen as a light source for actuation and spectroscopic analysis, using the haptic module to enhance mass transport in micro volumes, and the use of magnetic sensors are discussed.

## 1. Introduction

Personalized medicine aims to tailor medical treatment and patient follow-up to the specific needs of individuals [1]. This implies early diagnosis, timely monitoring of defined conditions, and the ability to provide specific treatment swiftly. Worldwide, but especially in societies with a growing and increasingly old population, global personalized medicine relies on the availability of efficient and inexpensive tools for POC and self-testing, which allow prompt result acquisition near the patient and also facilitate remote data analysis and interpretation for distant counseling and follow up. However, the current COVID-19 pandemic has revealed important weaknesses of our healthcare systems, stressing the lack of competitive instruments for personalized diagnosis [2]. The pandemic has exposed the challenge of carrying out population-wide testing in a very short time. Despite the wealth of diagnostic test kits in the market, these are, in general, addressed to professional users. Barring the exceptions of personal glucose meters and fertility and pregnancy tests, very few products are designed for the general public. This short review focuses on how mobile-smartphone-based POC technologies can facilitate universal access to advanced molecular diagnostics and transform the existing personal medicine landscape.

Broadly, POC devices are analytical tools performing molecular diagnoses outside the clinical laboratory. POCs should meet the ASSURED criteria laid out by the World Health Organization (WHO), which recommends that devices for POC testing should be Affordable, Sensitive, Specific, User-friendly, Rapid and robust, Equipment-free, and Deliverable to end-users [3]. Although these criteria were defined initially to fit the requirements of low-resource settings, such as in developing countries, the 2020–2021 pandemic has shown that ASSURED devices also make a difference in the most advanced societies. Clinical laboratories can carry out complex analytical work, such as antigenic, serologic, and nucleic acid testing [4,5], but access to them is limited and result delivery to the patient may take several days.

Of the different ASSURED criteria, “equipment-free” is perhaps the most important and hardest to meet. This is because reaching the extremely low detection limits —typically in the ng mL^−1^ range or lower—required for detection of most biomarkers involves sensitive, accurate, and highly reliable instrumentation. So far, only lateral flow and colorimetric tests are equipment-free [6]. These tests are designed to be used by virtually anybody with minimal or no formal training and are entirely manual. Consequently, the interpretation of their results is subjective, which may lead to errors, especially in cases near the detection limit, and data management relies entirely on the user, so the health system rarely receives the results in real-time, if ever [7,8]. In addition, their restricted sensitivity often limits both their analytical performance and their suitability for population-wide testing. Attempts to improve the performance of these tests have been made by incorporating measurement equipment—either customized readers, general-purpose optical detectors, or a smartphone camera [9]. Although at a higher cost, using a reader contributes to objectivize the result readout and might provide semi-quantitative and, frequently, more sensitive results.

**Figure 1:**
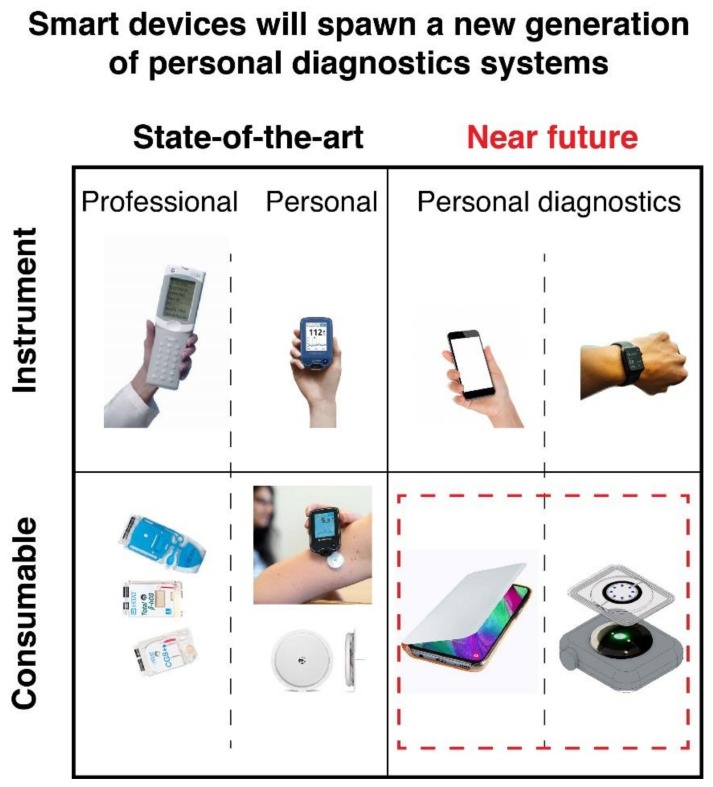
Point-of-care devices are two-part systems targeting professional and personal users. Mobile devices have the potential to replace dedicated instrumentation and lead to a new wave in personalized diagnostics. At the same time, the consumable part will adapt to this new paradigm. The left-hand side features Abbott’s i-Stat system and consumables, and Abbott Freestyle Libre and its biosensing capsule. The right-hand side illustrates the potential use of mobile devices, such as smartphones and smartwatches, to accomplish POC biosensing.

In general terms, the evolution of the variable and complex POC device landscape is driven mainly by three criteria, namely, (i) user profile, (ii) device features (functionality), and (iii) device configuration. Regarding user profiles and leaving application environments aside, Figure 1 distinguishes between two main user groups: the professional and the layperson. The latter forms the largest group, representing the greatest opportunity for new products, and, therefore, should receive attention and development effort more urgently. The second set of criteria is extremely complex because it addresses not only the ASSURED criteria but also analytical performance and other functional issues. Last, device configuration involves the “how”. It addresses how the device works, how and when it is used, and what happens to it afterward. Device configuration considers its components, materials, and fabrication processes and how the device interacts in its ecosystem.

Figure 1 also shows that, regardless of the user profile, quantitative POC devices consist of two parts with very different life cycles: a reusable instrument and a disposable consumable that receives the sample and where the detection takes place [10]. Among the mobile or hand-held instruments available for POC testing, smartphones stand out because they can be more than just optical readers and alarm systems. Their recent evolution has turned them into potential analytical instrumentation, as they can power, actuate, and be in full control of complex analytical procedures. While different POC manufacturers produce their own hardware and software instrument platforms, smartphones can make use of a large number of free applications for data analysis and can be connected securely to centralized databases to deliver results nearly instantly. One critical factor to successful personalized medicine is the timely management of diagnostic information. By their very nature, mobile devices favor the convergence of personal and professional systems and may eventually replace dedicated instruments and become a universal analytical platform.

## 2. Smartphone-Based Diagnostics Today: Optical Detection and Data Transmission

Today, mobile phone capabilities include 3G to 5G, WiFi, bluetooth, and near field communication (NFC). When mobile phones were first employed in POC settings in the early 2000s, users could only take advantage of their data transmission capabilities. They were, thus, initially used to replace personal digital assistants (PDAs) and facilitate communication between medical staff [11]. Mobile phone cameras began to be exploited shortly after, and one of their earliest uses was to capture screenshots from ultrasound images for sharing among professionals [12]. Shortly afterward, Whitesides et al. reported one of the first uses of a mobile phone camera to capture images from a paper-based assay and send them elsewhere for analysis [13]. Since then, the use of phone cameras has become widespread, and nearly all works reporting the use of mobile-phone-based diagnostic systems rely on image capture and analysis, as highlighted in recent reviews [14,15,16,17]. Mobile phone cameras have enabled colorimetric methods [18] and more advanced and sensitive techniques such as fluorescence [19] and electrogenerated chemiluminescence (ECL) [20]. Alternatively, the ambient light sensor (ALS) present in smartphones can also be used to develop simple optical sensors in tandem with light-emitting detection methods [21]. However, an important limitation of image-based systems is that they require bulky accessories to ensure controlled lighting or darkness and an adequate focal distance [22]. The silver lining is that some of the colorimetric tests available today will still render quantitative information if placed in direct contact with either the camera or the ALS, as already suggested by Hogan et al. [23].

Electrochemical detection methods, which are complementary to the aforementioned optical methods, can also be applied by mobile phones. Although working electrode potential control is difficult in solid-state devices, stable printed reference electrodes have been reported, showing that high-performance, inexpensive, and disposable devices can be produced [24]. Miniature potentiostats that can be connected to the mobile phone USB/charger port have been developed as well [25,26,27]. The audio jack has also been used to deliver power to an electrochemical cell. Although, in this case, potential control is restricted to potential steps, smartphones have been successfully used to drive an ECL-based detection system [20,23]. These works illustrate the feasibility of spectroelectrochemical analysis [28] using smartphones by coupling electrochemical actuation and optical detection. However, it can be anticipated that as mobile technologies evolve and audio jacks and charge connector ports disappear, electrochemical systems will remain very much alive [29,30].

## 3. Next Steps for Smartphone Full-Capacity Exploitation in Personal Diagnostic Systems

**Figure 2:**
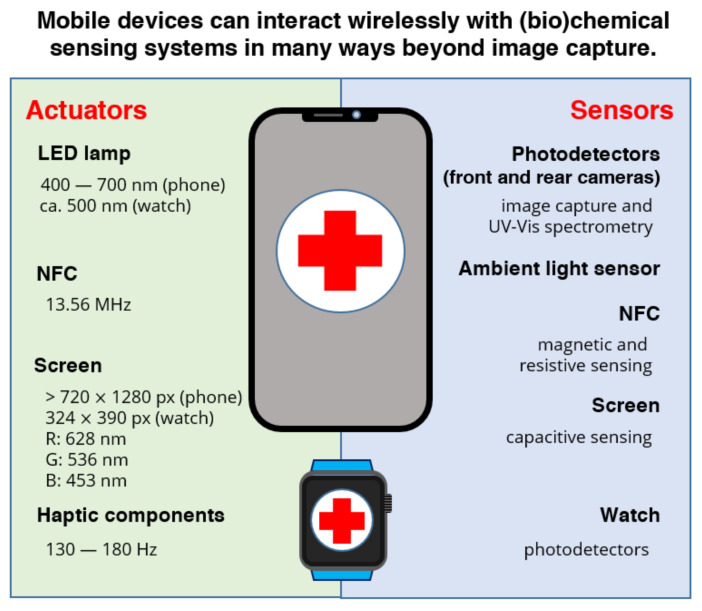
Summary of the main actuation and detection modes currently available in smart personal devices.

Besides image acquisition and data transmission, current smartphones display a variety of functions and capacities that can or could be helpful for the production of POC diagnostic devices, as represented in Figure 2. For instance, mobile phone devices can transfer energy wirelessly in three different ways, using radiofrequency (RF), light, and motion (vibration). RF is one of the easiest and most efficient ways to interact with the disposable component of the diagnostic device, as reflected by the increasing volume of publications reporting NFC-powered POC devices. The flashlight and the screen can be useful light sources too, and their use has been occasionally described. In contrast, haptic feedback systems responsible for providing a tactile response to certain user inputs, i.e., vibration, have received virtually no attention from the lab-on-a-chip community until now. This is despite the fact that they could facilitate reagent mixing in a smartphone-based POC or lab-on-a-chip device, for instance, by facilitating the dissolution of dry reagents in microchannels and microchambers upon wetting with capillary laminar flow.

### 3.1. NFC- and Smartphone-Based Diagnostics

Wireless chemical sensors and biosensors have attracted lots of attention because they can be installed and used anywhere in a few minutes without needing cables for power or communication; they have very low power consumption and they can transfer data dynamically for remote interpretation in nearly real-time [16]. Among the available alternatives, smartphones are cost-efficient devices for the development of wireless sensors. Most smartphones include different wireless communication protocols such as 3G to 5G, WiFi, bluetooth, and NFC. Of the different protocols, the ability of NFC to transfer data and energy efficiently [31] makes this technology a fantastic candidate to enable tomorrow’s personal diagnostic devices [32].

NFC technology was developed for contactless communications in 2002 [33]. The NFC communication process occurs between two compatible devices less than 10 centimeters apart [34,35,36]. Compared to other communication technologies such as bluetooth, which displays a setup time of about 6 s, NFC is around 60 times faster. There are two important issues to bear in mind when using NFC to control (bio)sensors. First, NFC antennas need to have similar coil shapes and sizes to ensure a good connection. Second, NFC is a 13.6 MHz alternate signal (AC). Although it is possible to couple NFC directly to sensors and biosensors [37,38], electrochemical (bio)sensors and other components operate in direct current (DC). Harvesting energy from an RF source typically involves some form of signal rectification. A rectenna, or rectifying antenna, is an antenna coupled to a rectifying element [39], the simplest of which may be a diode. While printed diodes may be used [40], they are typically slower and present higher forward voltages than conventional diodes, which, due to the timescales of the (bio)chemical processes involved, may not be too problematic.

NFC has found an increasing number of applications in the biomedical sector thanks to its unique ability to enable peer-to-peer communication between smartphones and NFC tags. These NFC tags can be battery-less or passive, requiring less power than bluetooth tags [41]. The NFC antennas can also be made stretchable and flexible for wearable and implantable devices [42]. This allows fabricating low-cost NFC tags and provides interesting power transfer and energy harvesting possibilities [43]. According to ISO/IEC 14443–2:2001(E), the minimum NFC unmodulated operating field is between 1.5 and 7.5 A m^−1^ rms (root mean square). The maximum power obtained is around 50 mW, enough to power sensors of very different kinds [30,35,44,45,46,47,48,49].

NFC systems are being used in different fields for many applications. Biomedical use includes the measurement of heart electrical activity, heart rate monitoring, and electrocardiogram, SpO_2_, endocrine pH, temperature and glucose sensing [30,35,44,45,46,47]. Focusing on the glucose measuring system reported by Fedtschenko et al. [45], the voltage requirement was 2.6 V for the analog part and 1.8 V for the digital part; the power consumption was less than 300 µW for the transponder and potentiostat. Thanks to these features, it was possible to measure from 0 to 20 mM glucose in tears. In another work, Teengam et al. [30] created a sensor powered by NFC for hepatitis B virus detection, obtaining good results compared with traditional methods. An NFC-powered biosensor has been reported for the wireless determination of cotinine in urine [38]. Another example of an NFC-powered biosensor is a wireless and flexible electrochemical skin patch for in situ analysis of sweat cortisol. This patch, which has a limit of detection ca. 7.3 nM cortisol, can reportedly cover the concentration range of sweat cortisol (22–286 nM) [50].

### 3.2. Light Actuation

**Figure 3:**
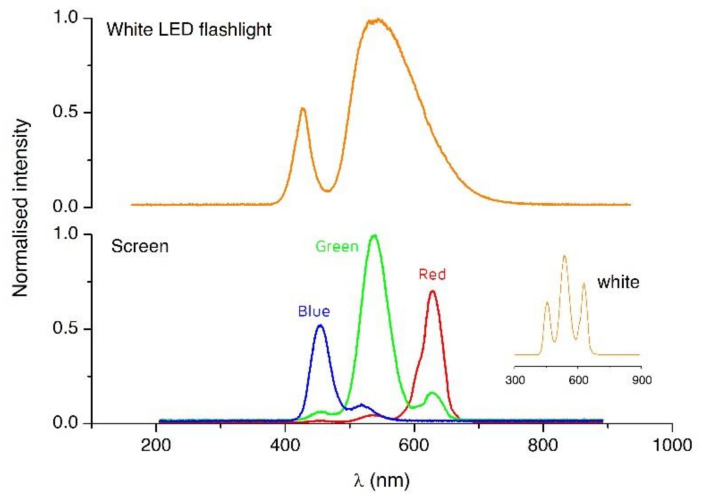
Light spectra from an iPhone 7. Top: LED flashlight. Bottom: individual spectra obtained from the screen emitting red (255,0,0), green (0,255,0), and blue (0,0,255) light. The inset shows the spectra obtained from a white (255,255,255) screen.

Other important power sources, but so far largely missed, are the flashlight and screen of smartphones, which have been tentatively used for detection in ELISA-like testing [51] and spectroscopic applications [22]. Figure 3 shows the spectra from the flashlight and the screen RGB pixels in a smartphone. The possibility of tuning the screen color offers intriguing and exciting actuation possibilities. For instance, photoactuated valves and regions of light-modulated surface tension have been integrated before using lab-on-a-chip technologies and can respond to light from a screen as well [52]. Although these photoactive materials are mainly responsive to UV light [53,54], similar materials that react in the visible range may be developed in the future, enabling the integration of comparable photoactuated valves and pumps [53,55], including channels where the flow is controlled by surface tension gradients on the channel walls. Light actuation may also be used to enhance detection through photoelectrochemical methods.

### 3.3. Haptic Feedback to Enhance Mass Transport

**Figure 4:**
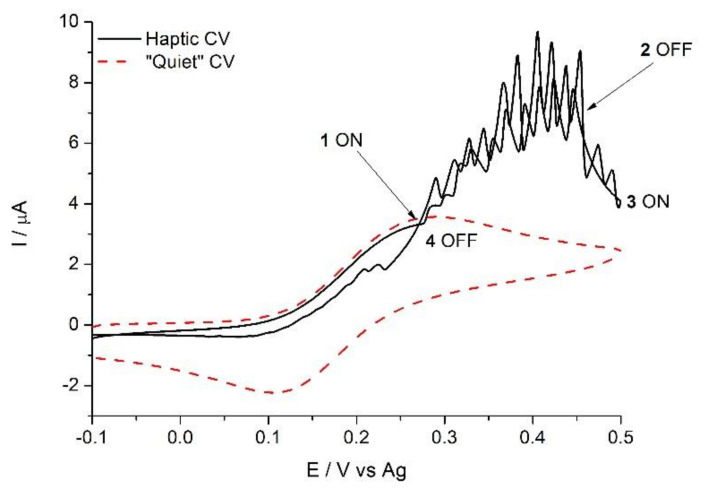
Haptic voltammetry. Cyclic voltammetry of 1 mM ferrocyanide in 0.1 M KNO_3_ at a 2.5 mm screen-printed graphite disk electrode (scan rate 25 mV s^−1^), shaken by a ringtone vibration (Haptic CV) and without vibration (quiet CV). The labeled points highlight the instant when the vibration starts or stops. Total solution volume was 60 μL.

Haptic stimuli, mostly used in smartphones as an alarm and user-communication system, may also be used to improve mixing in POC devices and improve their analytical performance. Haptic feedback is integrated into mobile devices using mainly eccentric rotating mass (ERM) motors that vibrate around 200 Hz. This frequency is close to the optimum 250 Hz picked up by the Pacinian corpuscles present in the skin [56]. However, this frequency is also well suited to enhance diffusion and increase the rate of transport-controlled processes, such as homogeneous reactions in microchannels and interfacial processes, and, particularly, electrochemical detection. Figure 4 shows what, to the best of our knowledge, is the first reported so-called “haptic voltammogram” (unpublished results). Turning on the haptic module of a smartphone, placed close to an electrode during detection, doubles the current registered at the electrode compared to the “quiet” case. Barring the differences in their mass transport coefficients, this voltammetry is reminiscent of sono-voltammograms [57]. This proof of concept shows the ability of smartphones to induce significant mixing in a microvolume in a controlled manner.

### 3.4. Magnetic Detection and Actuation

Magnetism finds many applications in biosensing, from sample preparation to detection and even actuation. Magnetic beads are now routinely used in bioassays and biosensors as they enable sample preconcentration and analyte extraction efficiently and effectively. However, the great majority of the magnetic-bead-based assays described involve external equipment, complex manipulation, or both [58]. Today’s phone compasses and smart cover detection rely on magnetic sensors based on the Hall effect or magnetoresistance. Smartphone magnetic sensor sensitivity is in the range of hundreds of nT, the Earth’s magnetic field being about 25–65 μT. Thus, the magnetic sensors on smartphones can precisely place disposable POCs featuring magnetic printed structures and even detect the presence and movement of magnetic beads. While no stand-alone POCs based on magnetic materials have been reported, magnetism offers many exciting possibilities for developing novel POC devices. Based on literature reports, magnetism can be partly exploited by mobile devices to detect immobilized magnetic markers to increase detection of large magnetic beads and to increase fluid dynamics in POC systems [59].

## 4. Bringing Molecular Diagnostics from the Clinical Lab to the Personal Sphere: Current Methods and Challenges for POC Implementation

Many of the examples described above demonstrate the ability of mobile phones to interface with lateral flow, paper-based and chip-based devices. This section provides an overview of laboratory-based methods that are currently challenging for mobile diagnostics.

### 4.1. Microscopy

**Figure 5:**
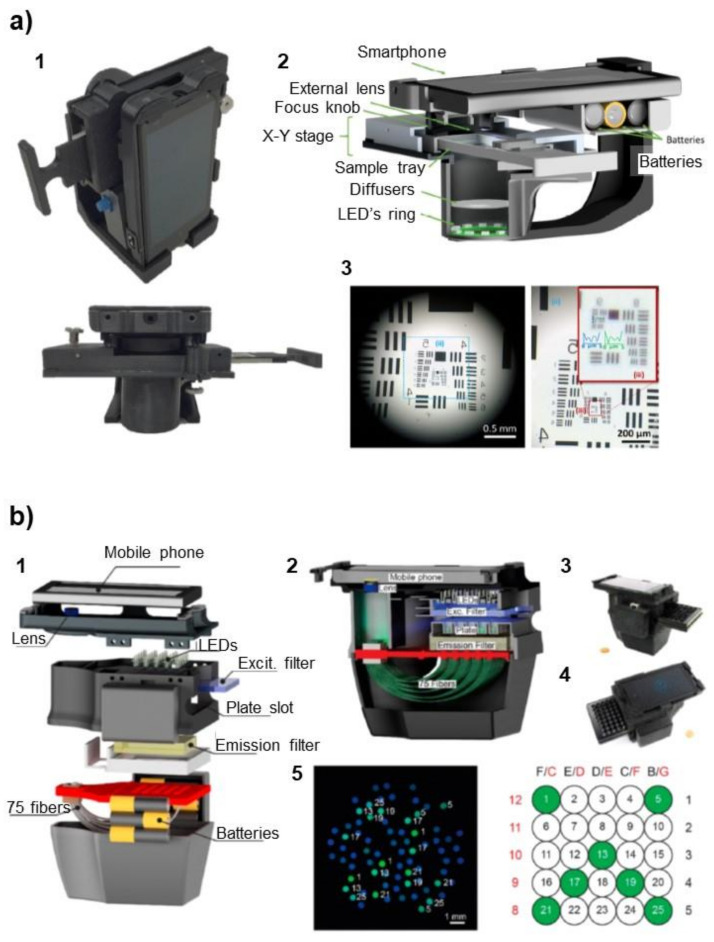
(**a**) Design of the smartphone microscope reported by Rivenson et al. 2018 (adapted with permission from [63]; copyright 2018 American Chemical Society). (1) Photography of the device taken from different views and (2) schematic illustration of its components. (3) Image of a USAF test resolution chart captured using the smartphone-based microscope. The smallest line width that is resolved is ~0.87 μm. (**b**) Design of the mobile phone fluorescence plate reader described in Kong 2017 for measuring LAMP DNA amplification carried in a 25-well plate (adapted with permission from [64]; copyright 2018 American Chemical Society). (1) Assembly of the mobile reader device and (2) schematic side view showing inner structure. The phone is assembled in a custom-designed 3D-printed optomechanical interface where the microtiter plate is inserted. An array of 5 × 5 blue LEDs was used as the excitation light source for fluorescence, filters were placed above and below the plate, and the fluorescence signal was collected by 75 optical fibers (3 per well) and imaged by the smartphone camera via a single lens. (3,4) Real photographs of the device. (5) Example of fluorescence image obtained by the random optical fiber pattern and corresponding well positions on the right.

Molecular methods based on microscopy rely on colorimetric, fluorescent, or luminescent detection methods, in most cases coupled to the staining of the sample or some of its components. Images obtained by microscopy are then interpreted by a well-trained professional or, more recently, using image analysis and machine learning. Although smartphone cameras have integrated increasingly sensitive photodetectors, they generally present poor magnification optics. Because of this, amplification strategies/accessories, such as the use of additional lenses [60] or light-emission-based detection methods [19,61,62,63], are required for the acquisition of acceptable images and data (Figure 5a). Microscopy is still, nowadays, the gold standard for certain types of measurements, such as the detection of *Plasmodium*, the parasite that causes malaria. While artificial intelligence cannot replace an experienced analyst, high-magnification optics will still be required, down to tens of microns.

### 4.2. Immunoassays

Enzyme-linked immunosorbent assays (ELISAs) provide accurate results but require multiple reagent additions and washing steps to ensure selectivity and an enzyme amplification step to increase sensitivity [65]. Attempts have been made to format mobile phones into hand-held microplate readers for ELISA result acquisition and interpretation [51,66]. Nevertheless, the multiple steps required to accomplish the whole test increase the overall assay time and relegate this method to centralized laboratories, where many ELISAs are automatized using sophisticated robotized platforms. The importance of ELISA cannot be overstressed as all immunoassays, including lateral flow assays, involve ELISA early in their development.

### 4.3. Nucleic Acid Testing

Multiple polymerase chain reaction (PCR) approaches exist for the amplification of nucleic acids (including nested conventional PCR, multiplex real-time PCR, and reverse transcriptase PCR) [5]. PCR is extremely sensitive, expensive, technically demanding, and prone to contamination and involves thermal cycles that need to be accurately controlled. In addition, PCR can fail to detect highly variable targets (such as viruses with high mutation rates) or low prevalence targets.

An interesting modality, which avoids thermal cycling, is isothermal amplification. Isothermal amplification has lower technical requirements and may be faster than classical PCR because temperature does not have to be cycled during each amplification step. For instance, loop-mediated isothermal amplification (LAMP) and isothermal thermophilic helicase-dependent amplification (tHDA) are conducted at a constant temperature of 65 °C, and nucleic acid sequence-based amplification (NASBA) works at 41 °C. Even if most isothermal amplification techniques require a complex design of primer batteries, multi-enzyme cocktails, and moderately skilled personnel, they are easier to automate than classical PCR using low-cost equipment; some examples have been already reported, in some of the cases using smartphones for data acquisition and interpretation [67,68,69]. In these examples, nucleic acid was amplified in a microfluidic chip, a magnetically actuated polymeric cartridge, or a 25-well plate in the presence of a fluorescence intercalating dye. End-point fluorescence was then measured using the camera of a mobile phone, which was placed in a customized holder containing a series of LEDs and optical accessories, such as filters, lenses, or mirrors (Figure 5b). Isothermal amplification is, thus, a good candidate for adaptation to mobile devices, but the device design must consider the working temperature because most mobile phones stop working above 45 °C.

### 4.4. Next-Generation Sequencing (NGS)

These methods entail the random amplification of large genome sections. They are used for the detection and characterization of highly changing microorganisms, such as the hepatitis B and C viruses, for which a single patient might display a range of closely related sequences that will determine the response to the treatment. NGS offers ultra-high throughput, scalability, and speed, allowing the generation of very large sequencing datasets, revealing many full genomes from individual templates at an increasingly affordable cost and in a short time [70,71]. NGS requires skills and expertise to analyze and interpret the large datasets generated using bioinformatics, which can also be carried out in the cloud [72]. Less technically demanding amplification-free methods have been developed by exploiting CRISPR, a bacterial adaptive immune system that targets specific single- and double-stranded DNA or single-stranded RNA substrates. In the example reported by Fozouni et al., the authors used a quenched fluorescent RNA reporter complexed to Cas13a nuclease. In the presence of SARS-CoV-2 genomic RNA with sequences complementary to the reporter, this was cut by Cas13a, releasing fluorescent fragments that were detected in real-time using a mobile-phone-based fluorescence microscope [73]. These results suggest that we can expect to see more adaptations in the future.

## 5. Conclusions and Outlook

Following the worldwide sanitary crisis of 2020, the need to develop population-wide testing strategies and tools has become clear and is more pressing than ever. Mobile devices are ubiquitous and have the potential to become truly personal analytical platforms in the near future. This is thanks to their powerful analog, digital, and telecommunication functions combined with cloud data processing. The new systems will combine the high-performance of advanced molecular diagnostics with the affordability of current lateral flow devices and will fulfill the ASSURED criteria more closely.

Here, we have shown examples of how mobile devices can be used beyond image analysis and data transmission to enable personalized diagnostic platforms that can reach a wider population base. Mobile devices can transfer different forms of wireless energy (radiofrequency, light, and even motion) to disposable sensing devices that are able to process a sample and return vital analytical information to the mobile device for further processing.

We believe that this vision is possible because both the technology and much of the required knowledge already exist. However, the development of disposable platforms integrating functionality beyond current lateral flow tests presents important challenges for both materials science and manufacturing, as the environmental impact and sustainability of the new platforms must also be considered.

## Data Availability

Data depicted in Figure 3 and Figure 4 are available on reasonable request to the corresponding authors.
